# Advances in Materials with Self-Healing Properties: A Brief Review

**DOI:** 10.3390/ma17102464

**Published:** 2024-05-20

**Authors:** Rashid Dallaev

**Affiliations:** Department of Physics, Faculty of Electrical Engineering and Communication, Brno University of Technology, Technická 2848/8, 61600 Brno, Czech Republic; rashid.dallaev@vut.cz

**Keywords:** self-healing, polymers, crack healing, microcapsules, implants, hydrogels, ceramics, concrete

## Abstract

The development of materials with self-healing capabilities has garnered considerable attention due to their potential to enhance the durability and longevity of various engineering and structural applications. In this review, we provide an overview of recent advances in materials with self-healing properties, encompassing polymers, ceramics, metals, and composites. We outline future research directions and potential applications of self-healing materials (SHMs) in diverse fields. This review aims to provide insights into the current state-of-the-art in SHM research and guide future efforts towards the development of innovative and sustainable materials with enhanced self-repair capabilities. Each material type showcases unique self-repair mechanisms tailored to address specific challenges. Furthermore, this review investigates crack healing processes, shedding light on the latest developments in this critical aspect of self-healing materials. Through an extensive exploration of these topics, this review aims to provide a comprehensive understanding of the current landscape and future directions in self-healing materials research.

## 1. Introduction

In response to the growing demands for increased durability, reliability, and safety, composite materials with self-healing abilities have been developed, drawing inspiration from the innate healing abilities observed in plants and animals. These self-healing materials (SHMs) have found widespread application in aerospace, marine, biomedical, and structural fields, with advancements extending to numerous other domains. In the works [1,2,3], the applications of self-healing polymers and nanocomposites, along with their recent developments across various sectors are discussed, offering insights into product-based outcomes and future prospects of these materials. The ability of artificial materials to self-heal any properties can increase their service life, reduce the cost of maintaining them in working condition and repairs, and also increase the level of safety of the structure or product as a whole. For this reason, SHMs are currently the subject of one of the most researched areas of materials science [4,5,6]. The advancement of self-healing effects finds its pinnacle in polymer materials, owing to their capacity to swiftly restore not only intermolecular bonds but also, under specific conditions, generate new ones during the cross-linking process. The presence of cross-links within polymers dictates relatively elevated diffusion rates, while the nature of interactions (covalent versus non-covalent) determines the mechanism for consolidating damaged boundaries and reestablishing bonds.

Self-healing is the capacity of a material to naturally and autonomously recover from damages without external interference. Various terms like self-repair, autonomic healing, and automatic repair are used to describe such characteristics in materials. Some products necessitate external intervention to initiate self-healing properties [7,8], resulting in two modes of self-healing processes: autonomic (no external intervention required) and non-autonomic (requires human or external triggering). The effect of self-healing (self-healing) in artificial materials is a complete or partial reduction in the surface area of damaged material due to mass transfer and combining boundaries (consolidation) with full or partial restoration of the functional characteristics of the material.

### 1.1. Applications of SHMs

SHMs present a diverse array of applications, notably exemplified by self-healing fabrics capable of repairing punctures or tears at room temperature. The healing process is effortlessly triggered by a gentle rubbing action near the affected areas, eliminating the need for external agents. Moreover, these materials boast exceptional stretchability, able to elongate to more than half of their original length, while maintaining resilience to stress when under tension.

Innovation in SHMs extends to the electronics sector, where LG Corporation (LG Group) is pioneering the development of mobile phones with SHM coatings [9]. These coatings fortify the devices, preventing breakage upon impact from certain heights, while also featuring scratch-resistant displays for extended longevity. Additionally, electronic equipment, circuit boards, and wires constructed with SHMs offer reduced maintenance costs and prolonged lifespans. Furthermore, self-healing ceramics provide scratch-free ceramic floor tiles, preserving both their aesthetic appeal and durability over time.

The resilience of self-healing metals is particularly noteworthy, especially in high thermal stress and corrosion conditions. In the construction industry, the integration of SHMs in concrete is becoming increasingly prevalent, with various techniques employed to embed self-healing mechanisms within concrete structures [10]. Healing-based capsules, pre-filled within the concrete during construction, stand ready to mend any cracks that may develop over time, thereby promoting the longevity and structural integrity of the construction [11].

**Figure 1:**
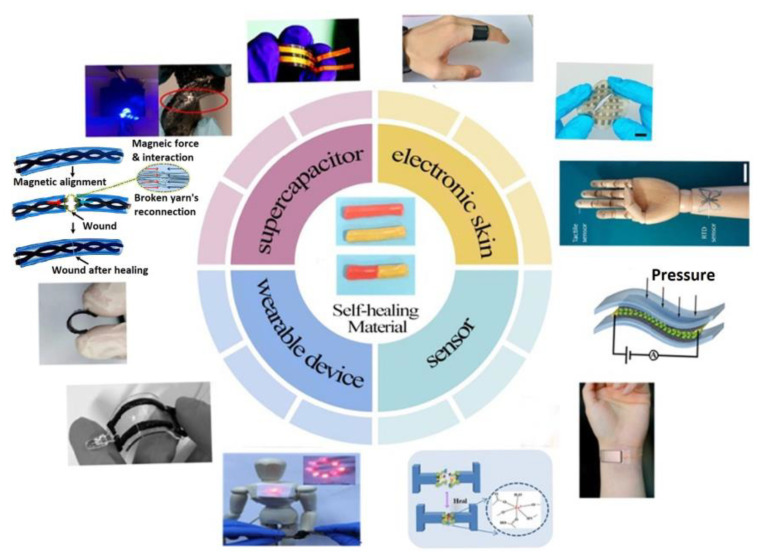
Applications of SHMs [13] (Permission to use was granted by Elsevier).

The multifaceted utility and practical significance of polymer materials, characterized by their ability to retain shape, respond to stimuli, and self-heal, underscore their immense potential in the burgeoning field of materials science. Conceptually promising projects aimed at developing “smart” SHMs offer innovative solutions to materials science challenges [12]. Moreover, polymer-based SHMs hold significant promise across a spectrum of practical applications, including batteries, wearable devices, electronic skin, sensors, and supercapacitors. Figure 1 illustrates the prominent applications of SHMs.

### 1.2. Self-Healing Mechanisms

The authors of [14] explore various mechanisms within the realm of self-healing, targeting the restoration of materials damaged by cracks that compromise their mechanical integrity. Even minute pinholes can undergo filling and restoration processes, ultimately bolstering the mechanical performance of the materials. Inspired by biological systems, the development of this innovative class of smart materials takes cues from nature, where many materials exhibit inherent self-healing properties. In essence, self-healing materials (SHMs) possess the innate capacity to substantially regain their mechanical properties post-damage. This recovery process can occur autonomously or be triggered by specific stimuli like heat, radiation, or pressure. As a result, these materials are poised to significantly enhance the safety and durability of polymeric components without necessitating costly active monitoring or external repairs [15].

In self-healing materials (SHMs), the process of “healing” relies on the consolidation of damaged boundaries, which occurs subsequent to their reduction through mass transfer. Mass transfer, within the context of self-healing materials, involves the movement or diffusion of healing agents throughout the material to mend damage. When a self-healing material experiences damage, such as cracking or fracturing, the healing agents housed within it are released and conveyed to the site of damage via mass transfer mechanisms. This transport mechanism can take various forms, including diffusion, capillary action, or flow within the material matrix. Upon reaching the damaged area, the healing agents react or recombine to restore the material’s integrity, effectively repairing the damage. Mass transfer is pivotal in enabling the self-healing capacity of these materials by facilitating the transport of healing agents to the areas in need of repair [16].

These processes can occur autonomously, such as through the flow of material, or non-autonomously, when healing is triggered by external influences like increased temperature or ultraviolet radiation. Self-healing mechanisms in artificial materials are categorized into “external” and “internal” based on how the healing process is organized. The “external” mechanism relies on restorative components embedded within the base material’s matrix, such as microcapsules containing healing substances (discussed further in the next subchapter), while “internal” self-healing mechanisms operate without the need for additional restorative compounds [17].

**Figure 2:**
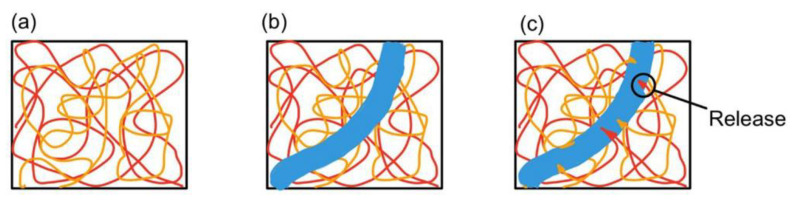
(**a**) The polymer composite matrix contains a vascular network, (**b**) depicted by the blue region, where a cut is made, and (**c**) monomers from the microchannel seep into the matrix [19]. (The figure is available in Open Access).

In contrast to the microcapsule self-healing method, the vascular network self-healing system does not rely on storing healing agents within capsules. Instead, the healing agents are housed within microchannels designed to mimic the structure of blood vessels found in the human body, as demonstrated in a biomimetic vascular network pioneered by C. Dry [18]. This approach, illustrated in Figure 2, operates on the principle of a vascular network facilitating self-healing processes.

Dynamic covalent chemistry, encompassing reactions like imine formation, boronate ester complexation, catechol-iron coordination, Diels–Alder reaction, and disulfide exchange, plays a significant role in crafting self-healing hydrogels. These bonds exhibit a stronger yet slower dynamic equilibrium compared to non-covalent interactions [20].

### 1.3. Composite SHMs and Microcapsules

The development of composite systems based on SHMs makes it possible to use and improve the self-healing characteristics of the materials from which they are composed. The introduction of various fibers, chemical components, etc., reduces the degree of destruction of the original material and also accelerates the complete healing of the defect. Thus, the presence of elastic fibers in the matrix helps to reduce the boundaries of the damaged area of the polymer after deformation, accelerating the healing process. A large number of works are devoted to the study of materials with inert, fragile capsules with a healing substance introduced into the main matrix [21,22,23]. When a defect occurs, the capsule breaks, releasing a healing agent that spreads to the site of the defect. At the same time, it either interacts with the matrix or the external environment, or is mixed with a catalyst—a hardener, also embedded in the matrix, hardens and restores the damaged area [22,24,25,26]. The design development of new materials based on the genetic code has entered the stage of active implementation, having a serious impact on our lives. The functional characteristics of materials capable of autonomously detecting damage and repairing it after complete destruction of the structure provide long-term performance characteristics for products based on them. Artificial “self-healing” materials would open up enormous possibilities, especially in cases where the reliability of materials needs to be ensured for as long as possible in difficult-to-reach areas [27]. 

In the literature, one can find a description of SHMs with microcapsules that act as a healing agent when the microcapsule of a polymer material is destroyed, both with the introduction of a thermoplastic into a polymer matrix and the use of the principle of hydrogen bond rearrangement. Thus, the authors of the work [15] describe synthesized acrylic copolymers with introduced carbon nanotubes. The introduction of nanomodifiers makes it possible to increase the elastic modulus and strength of the copolymer. External self-healing mechanisms, considered as autonomous self-healing, are usually classified based on the type of storage vessels used, although the concept of self-healing is similar. Capsule-based self-healing and vasculature self-healing are two types of extrinsic self-healing mechanisms.

In [28], a mechanism is used in which a mixture of monomers and a photoinitiator of the polymerization reaction are encapsulated in silicon dioxide microcapsules. Taking into account the high thermal stability of silicon dioxide, such materials have great prospects for use in the aerospace industry. To impart self-healing properties to materials, filled microtubes are used. In [29], the authors present an elastomeric composite containing hollow glass microtubes, which are filled with a healing system containing an alkyne, a thiol, and a photoinitiator. When such material is damaged, photoactive healing agents are released from the tube and, under the influence of ultraviolet radiation, the polymer crosslinks at the site of damage.

Another example of capsule systems suitable for space applications is a material developed by the Smart Materials and Sensors for Space Missions Division of MPB Technologies (Montreal, Canada), which is intended to protect against impacts from micrometeoroids and space debris particles [30,31].

**Figure 3:**
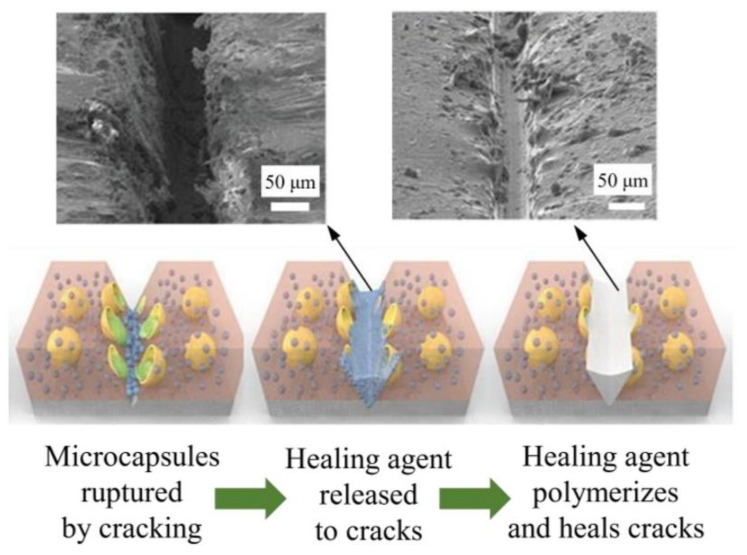
The self-repair mechanism triggered by microcapsules [32] (The figure is available in Open Access).

The primary self-repair mechanism in microcapsule-based materials involves two key steps: (i) as cracks develop, they rupture nearby capsules, and (ii) the released rejuvenator flows into the cracks to effect repairs. Specifically, these microcapsules, containing rejuvenating agents, are embedded within building materials [32]. Figure 3 depicts the self-healing process in polymer coatings, which is utilized here to demonstrate the general self-repair process involving microcapsules, applicable across various materials such as asphalt or cementitious mixtures.

### 1.4. SHMs in Biomedicine

The discovery of the basic laws of polymerization and polycondensation of organic compounds with the subsequent development and industrial production of synthetic polymers marked the beginning of their widespread use in medicine. Currently, modern reconstructive surgery of the heart and blood vessels (replacing defects in the walls and septa of the heart, providing artificial circulation) is unthinkable without polymers [33]. In radiation therapy, an important aspect of therapeutic and diagnostic measures is the use of elastic and easily formed materials, which ensure the safe and reliable mounting of radiation sources on the patient’s body and their targeted transportation to the treatment object [34].

The range of biomedical materials that have managed to reach the level of clinical implementation is steadily expanding. These materials include, for instance, polymers such as polyvinyl alcohol (PVA), polyacrylamide, and polyethylene glycol (PEG) [35]. Recently, superelastic alloys with a shape memory effect have attracted the close attention of researchers and clinicians. Developments in this direction lie in related areas of various sciences at the intersection of medicine and technology and affect the interests of representatives of various specialties—from physicists and engineers to practicing doctors. An important feature of today’s medicine is the increased importance of the quality of treatment. This largely determines the progress in the field of medical equipment. The development and implementation of new-generation bioinert materials and original designs made from them are becoming an integral feature of modern medical materials science and medical technology. New long-term functioning products and devices that are similar in behavior to body tissues meet a higher level of medical and technical requirements than “conventional” materials and designs [36].

### 1.5. SHMs in Implants and Prostheses

While current artificial systems still fall short of emulating biological skin and analogs, a similar healing approach is emerging, known as the “vascular system”. Analogous to the circulatory system in living organisms, this approach relies on pumps to circulate “healing” components through a network of “vessels”. These vessels can take the form of both 2D and 3D vascular systems, with various configurations available. Self-healing occurs through the simultaneous rupture of fibers (“vessels”) containing different reagents, which, upon mixing, undergo hardening akin to two-component epoxy resins [37,38,39].

In recent years, there has been a significant increase in the number of polymers used for the production of medical devices, particularly tubing [40,41]. Examples of such polymers include poly(ε-caprolactone) (PCL), polyether ether ketone (PEEK), and polyurethane (PUs), which exhibit a shape memory effect where the material regains its original shape at the defective area. Medical-grade materials must possess good resistance to surrounding tissues and body fluids, as well as high physical and mechanical properties, and chemical resistance to sterilization agents [42,43].

Ultra-high molecular weight polyethylene holds a special place among polyethylenes, being one of the optimal materials for cup liners for joint endoprostheses [44]. From an environmental standpoint, polyethylenes are considered harmless as they do not release hazardous substances into the environment. They find application in various medical devices such as packaging and adhesive films, catheters, drainage and irrigation devices, support plates for semi-permeable membranes in hemodialyzers and heme oxygenators, connecting elements, syringe tubes, droppers, and laboratory glassware [45].

Modern surgical practice widely utilizes various types of implanted foreign bodies to replenish structures or functions lost due to injury or disease. These include suture materials, prostheses, and technological devices aimed at restoring impaired organ functioning. This review [46] focuses on Russian import-substituting products, offering comparative characteristics and parameters of implants, highlighting their advantages and disadvantages, modern development approaches, and the requirements placed on them by practical surgeons. Implants should mainly perform the strength functions of the tissues and organs being replaced, at least in the immediate postoperative period [47].

General requirements for polymer implants:

- Biocompatibility (ideally bioinert);
- Certain physical and mechanical properties;
- Resistance to infection;
- Ability/resistance to biodegradation;
- Minimum material consumption;
- Ease of use;
- Economic accessibility.

## 2. State-of-the-Art

Undoubtedly, SHMs have many advantages. One of the most notable is the development of realistic artificial manipulators and other types of soft robotics. Now, as part of Project SHERO, researchers at the University of Cambridge have created low-cost salt and gelatin materials that can sense strain, temperature, and humidity using soft sensors and self-heal at room temperature [14]. This discovery is set to revolutionize the field of robotics and perhaps some other fields. These new materials differ from their previous counterparts in that they do not need to be heated to heal themselves. They can autonomously (without human intervention) detect the extent and location of damage, then self-regenerate and return to work again.

A group of scientists from the University of Texas at Austin, USA, under the leadership of Yu. Guihua, has created a flexible electrical circuit based on a special gel, which, if cut into two parts, is completely self-healing and resumes its original electrical conductivity. The new gel has a combination of properties that previously have never been seen together, these are flexibility, high electrical conductivity, and the ability to self-heal at room temperature. This solution opens up a wide range of possible applications: flexible electronics, robotics, electric batteries, and even soft artificial skin and biomimetic prostheses [48]. 

Singaporean researchers have invented a new foam material, AiFoam, which mimics skin for a robotic prosthesis. The main features of this material are the ability to regenerate and the ability to transmit tactile sensations. According to scientists from the National University of Singapore, the foam is created by mixing a fluoropolymer with a compound that reduces surface tension. This allows the artificial leather to literally “heal” damage—the foam itself fills cuts and other voids. As the inventors of the material note, it can be used both in robotics and to create high-quality prosthetics [49].

The creation of new artificial “self-healing” materials with a certain set of physical and chemical properties is steadily growing. The industry produces several types of polymers that meet basic medical requirements. These include polylactides (for implants of various types), ultra-high molecular weight polyethylene (for joint endoprostheses) [50], polyamides (for surgical threads), polyurethanes (for artificial heart chambers) [51], silicone polymers with high chemical and physiological inertness and thermal stability (for cosmetic surgeries on the face and mammary glands, the manufacture of catheters, heart valves, films to protect the skin surface during burns) [52], polyisobutylene in combination with natural polymers (adhesive compositions), polyparaxylene (for suture materials), polyacrylates (for use in bone grafting as tubes for drainage of the lacrimal sac, maxillary cavity, prosthetic blood vessels, heart valves, esophagus, stomach, bladder, bile ducts, urethra, eye lens; pins and plates for fixing bones during fractures, polymer mesh “frames” for connecting intestines, tendons, trachea, etc.). Particularly high demands are placed on polymers and composites for orthopedic dentistry and maxillofacial surgery [53].

Prostheses made from polyester fibers have been successfully used for more than 20 years to replace damaged areas of the vascular system. The material is used to produce blister packaging for instruments, surgical threads, synthetic blood vessels, and implants [54]. In some cases, antimicrobial and multilayer implants with anti-adhesive (anti-adhesive) properties are of particular interest [55,56]. Endoprostheses made of polypropylene monofilaments are currently the most common. Polypropylene has high biological inertness and resistance to biodegradation [57]. Of the acrylic polymers, polymethyl methacrylate (plexiglass or plexiglass) has found the greatest use in medicine—for optical systems of endoscopes [58] structural elements of medical devices [59], spectacle and contact lenses [60], droppers for blood transfusion systems [54], prostheses [61], preservation containers [62] dentistry (artificial jaws teeth and fillings) [61,63].

Studies on the implantation of various materials into the body have proven that it is polyurethane foam that “takes root” best. At the same time, an inflammatory process is observed in the tissues, during which granulation tissue is formed in the pores of the elastomer-young, rich in blood vessels and necessary for healing. Today, polyurethane is used to relieve a wide variety of health problems.

Promising barrier agents that effectively prevent intra-abdominal adhesion are gels made from absorbable polymers [64,65]. The gel is able to separate desulfurized surfaces for the time necessary for their remesothelization.

Self-healing hydrogels are of particular interest due to their high water content and controlled rheological properties [26,66]. Due to these properties, self-healing hydrogels mimic the extracellular matrix, making this class of smart polymers competitive candidates for biomedical applications [20]. Hydrogel layered composites, each layer of which has different sensitivity, make it possible to create new types of sensors, membranes, etc. based on them [67]. Hydrogels are proposed to be used in medicine as biocompatible materials [68], for example, for drug delivery systems [69,70,71] or bactericidal coatings of medical instruments [72]. Self-healing hydrogels are three-dimensional chemical or physical reversible networks that can restore the original morphology after damage. Dynamic bonds dominate the processes of dissociation and recombination and impart self-healing properties similar to the restoration of human tissues [20]. The best anti-adhesion effect was obtained using gels based on cellulose ethers (methylcellulose, sodium salt of carboxymethylcellulose, etc.), which have high biological inertness [73]. 

An aggravating factor complicating recovery after surgery may be caused by the formation of adhesions. Taking into account the etiological factors, a wide variety of anti-adhesive agents (barriers) in the form of membranes, films, and gels are considered to prevent and reduce postoperative adhesions and eliminate the mechanisms of their formation.

The gel acts as an artificial temporary “barrier” between damaged serous surfaces, ensuring their effective separation during healing, and then dissolves. Reducing the adhesion of the surfaces of organs and tissues helps maintain their mobility and prevents the formation of adhesions [74,75].

The tunable properties and environmental responses of smart polymer materials provide the opportunity to develop personalized biomedical products. The authors of [76] focus on three typical polymer smart materials, including stimulus-responsive, self-healing, and shape memory materials. The review also discusses some recent applications in precision medicine, such as 3D bioprinting, cell therapy, and tissue engineering The unique mechanism of regeneration of a number of materials opens up great prospects for further development, depending on the solution to the assigned problems: modeling the structure of the material will open up the possibility of obtaining composites with the properties of this effect (self-healing, self-healing), which are unattainable in other materials.

At the stages of design and development of products made from polymer materials, comprehensive materials science analysis acquires particular importance. Toxicological assessment of polymer materials used in medicine under conditions of direct contact with a living organism is important [77].

There are compounds that are derived from polyorganosiloxanes (silicones, siloxanes) containing the Si-O-B group. These compounds are sometimes referred to as “BS” compounds due to the presence of boron (B) in their chemical structure. Mechanically, BS behaves as a non-Newtonian fluid, exhibiting fluidic properties under static loads and elasticity under short-term or shock loads. This unique characteristic enables BS-based materials, when integrated into composite systems under low-speed loads, to facilitate mass transfer to the damaged area and affect the healing of defects [78]. Notably, the properties of this nanomaterial, which serves both protective and structural roles, bear resemblance to the biological process of blood clotting.

Materials featuring covalent bonds exhibit greater strength, as reduction occurs through cross-linking reactions (e.g., Diels–Alder). Consequently, the incorporation of microinclusions becomes necessary to facilitate cross-linking and substance healing, albeit imposing constraints on the material’s longevity due to the gradual depletion of the introduced substance. Conversely, repairing damage via non-covalent interactions (such as the formation of hydrogen bonds, complex compounds, ionic interactions, and van der Waals forces) is characterized by facile bond rupture and restoration, thus amplifying the potential for repeated healing and, consequently, enhancing material durability. However, such systems are sensitive to reduced loads and temperatures [22,25,26,79].

In contrast to covalent bonds, weak interactions such as hydrogen bonds offer greater potential for creating SHMs. A remarkable example of such an autonomous self-healing polymer is an oligomeric thermoplastic elastomer. Upon damage, simply pressing the fractured surfaces together allows the material to regenerate [80,81].

Another type of material that should be mentioned is photofluidic materials. Photofluidic materials refer to substances or composites that exhibit changes in fluidic behavior, such as flow or viscosity, under the influence of light. These materials are often engineered to respond to specific wavelengths or intensities of light, enabling precise control over their fluidic properties [82].

For the development of wearable ultraviolet (UV) detection technologies, photochromic materials have garnered significant attention lately. These materials offer the advantage of not needing electronic components, leading to systems and devices that change color upon irradiation. Their application in wearable technology, however, is currently constrained by the properties of the materials, particularly in meeting requirements for lightweight, compliance, and durability, especially under mechanical stress [83].

In [84], the authors fabricated a photochromic elastomer composed of diarylethene, PDMS, and toluene which demonstrated dual capabilities in self-formation and healing. These attributes were harnessed to realize signal transmission dependent on pulse frequency. Given that the device operates without necessitating high-powered pulses or ultra-fast signal sources, it holds promise for the development of an efficient smart signal transmission system.

## 3. Methodology and Relevance of the Review

### 3.1. Methodology

The methodology employed in conducting the review paper titled “Advances in Materials with Self-Healing Properties: A Review” involved a systematic and comprehensive literature search to identify relevant studies, research articles, review papers, and patents related to self-healing materials (SHMs). The search was conducted using academic databases such as PubMed, Scopus, Web of Science, and Google Scholar, utilizing keywords such as “self-healing materials”, “self-repairing polymers”, “self-healing composites”, and “autonomous repair mechanisms”.

The inclusion criteria for selecting studies encompassed publications that focused on recent advances, developments, and innovations in materials with self-healing properties across various fields, including but not limited to aerospace, automotive, construction, biomedical, and electronics. Both experimental studies and theoretical analyses were considered, with a preference for peer-reviewed articles published within the last decade.

Upon identification of relevant literature, the retrieved articles were screened based on their relevance to the review topic, and duplicates were removed. The selected articles underwent thorough examination and analysis to extract key findings, methodologies, experimental techniques, and future implications related to SHMs. Additionally, citation tracking and reference chaining techniques were employed to identify additional relevant studies not captured in the initial search.

### 3.2. Relevance 

SHMs have garnered significant attention in recent years due to their potential to revolutionize various industries by enhancing the durability, reliability, and safety of structural components. As the demand for advanced materials capable of autonomously repairing damage continues to grow, understanding the latest developments and emerging trends in SHMs is crucial for researchers, engineers, and industry stakeholders. A comprehensive review of recent advances in materials with self-healing properties is essential to provide insights into state-of-the-art technologies, identify key challenges, and explore future directions for research and development in this rapidly evolving field.

Despite the rapid progress in the field of SHMs, there remain several knowledge gaps and unresolved challenges that warrant further investigation. While numerous studies have focused on developing novel self-healing mechanisms and materials, there is a need for a systematic review that gathers relevant information and noteworthy results from existing literature and identifies the most promising approaches. Additionally, the scalability, cost-effectiveness, and environmental impact of self-healing technologies require deeper exploration to facilitate their widespread adoption in real-world applications. Furthermore, the integration of SHMs into existing infrastructure and manufacturing processes presents unique engineering and design challenges that need to be addressed. By addressing these knowledge gaps, future research endeavors can contribute to the advancement and commercialization of SHMs for diverse applications.

## 4. Experimental Data on SHMs

### 4.1. Self-Healing in Polymers

Self-healing polymers represent a classic category of smart materials capable of autonomously restoring their structure and original functionality following repeated damage [85]. These materials, known as “self-healing” substances or systems, are engineered to partially or fully recover their initial characteristics after sustaining damage, ideally without requiring external intervention or particularly human involvement [26,86]. In nature, self-healing phenomena manifest at various scales, ranging from molecular-level repairs, such as DNA restoration, to macroscopic processes like the healing of fractures and damaged blood vessels. Recent studies by authors [87,88] demonstrate the efficacy of producing self-healing Phase Change Materials (PCMs) by incorporating thermoplastics into the polymer matrix, utilizing methyl acrylate copolymers as the thermoplastic healing agents. This innovative approach obviates the need for monitoring reaction completeness, and the healing agents do not have expiration dates. Encapsulation of healing agents can be used not only in the PCM industry but also for protective coatings of metals, where the top layer, consisting of fibers, acts in the same way as therapeutic capsules in a polymer matrix [89].

Despite the higher cost associated with polyurethane, its remarkably brief curing time could potentially justify the expense by minimizing downtime [90], ensuring swift preparation enables rapid coating of the substrate, minimizing downtime to maintain continuous substrate protection. However, delayed healing of microcracks can lead to unintended side reactions on the fracture surface, such as disulfide bond reduction or hydrogen bond saturation, hindering subsequent self-healing processes. Therefore, expediting the self-healing process and improving its efficiency are paramount. Consequently, the capacity of polyurethane to self-heal quickly while achieving satisfactory mechanical strength recovery is crucial for mitigating substrate corrosion [91]. All small scratches that appear on the surface disappear. The self-healing film also repels dirt and water, so gadget owners do not have to worry about streaks and greasy fingerprints appearing on the display [92].

The self-healing effect can be realized in various types of materials, both in “pure” substances (polymers and prepolymers, ceramics, cements, and metals) and in complex composite systems (reinforced, layered, encapsulated materials, systems with fibers, vascular systems, sandwich panels with liquid reagents, etc.) [22,24,25,26].

Self-healing polymer composites represent a completely new class of materials endowed with the ability to regenerate. They are able to independently repair minor mechanical damage due to their structure. Today, there are two fundamental methods for producing self-healing composites: with and without admixtures. In the first case, special healing additives are used in the form of spherical capsules or tubes. The polymer is a material in itself, initially well adapted to the introduction of various additives into it. To ensure self-healing, thin-walled inert fragile capsules with a healing substance are introduced into it [93,94].

**Figure 4:**
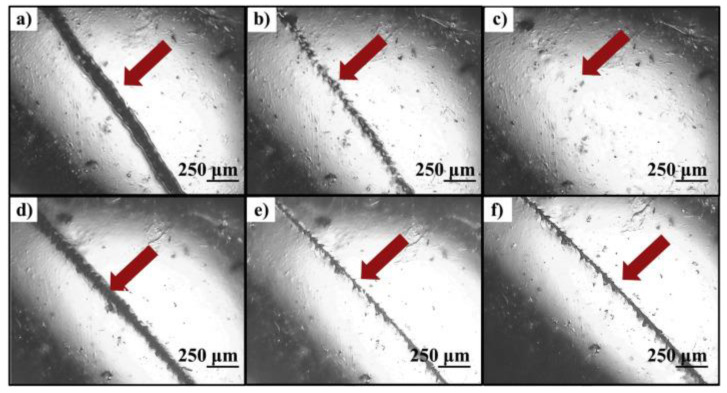
The self-healing experiment conducted on copolymer P5, with visualization using a microscope. Panels (**a**–**c**) depict healing experiments performed at 140 °C, showcasing the scratch before annealing (**a**), after annealing for 2 min (**b**), and after annealing for 5 min (**c**). Panels (**d**–**f**) display healing experiments conducted at 80 °C, showing the scratch before annealing (**d**), after annealing for 2 h (**e**), and after annealing for 48 h (**f**) [95]. (Permission to use was granted by Elsevier).

Figure 4 illustrates the self-healing studies conducted on copolymer P5 (butyl methacrylate). The scratch was completely healed at 140 °C within 5 min and nearly disappeared after 48 h at 80 °C. Similarly, copolymer P6 (poly(2-(dimethylamino)ethyl methacrylate) exhibited self-healing behavior at temperatures exceeding 120 °C within a 5-min timeframe. However, the surface of the coating appeared less smooth compared to copolymer P5. Both copolymers P5 and P6 demonstrated superior self-healing properties in terms of scratch size and lower healing temperatures.

**Figure 5:**
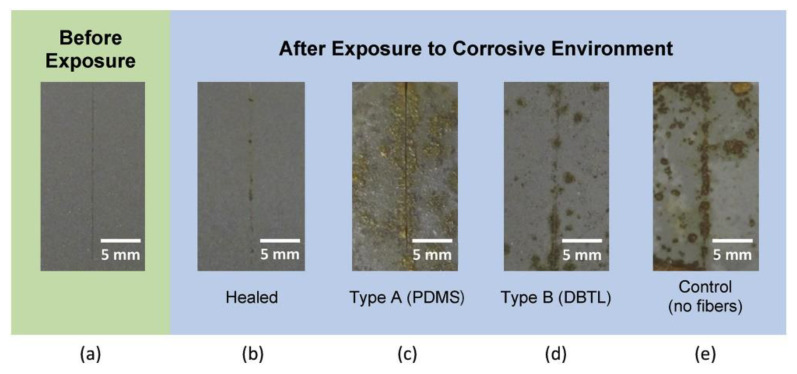
Optical images of both healed and control coatings captured four months post-exposure to a corrosive aqueous salt solution during electrochemical characterization: (**a**) before exposure, (**b**) healed, (**c**) Type A (PDMS), (**d**) Type B (DBTL), (**e**) Control (no fibers). Comparing the healed coating before and after corrosion exposure reveals similarity in appearance. Conversely, the control cases comprising type A (PDMS) fibers only, type B (DBTL catalyst) fibers only, and no fibers (silicone binder only) exhibit unmistakable signs of corrosion damage [89] (Permission to use was granted by Elsevier).

Furthermore, in [89] the authors demonstrate the healing process of polymer coatings after being exposed to a corrosive environment. The coatings, both the self-healed and control ones, were visually inspected after being subjected to a corrosive aqueous salt solution during electrochemical testing. Optical images of the coatings taken four months post-exposure to the corrosive environment are depicted in Figure 5. Before the electrochemical analysis, the scribed coatings showed no visible signs of corrosion. However, subsequent exposure to the corrosive environment resulted in significant corrosion damage and undercutting in the three control cases: type A (PDMS) fibers only, type B (DBTL catalyst) fibers only, and those with no fibers. Conversely, the healed coating exhibited minimal signs of corrosion, primarily localized to the scribed region.

**Figure 6:**
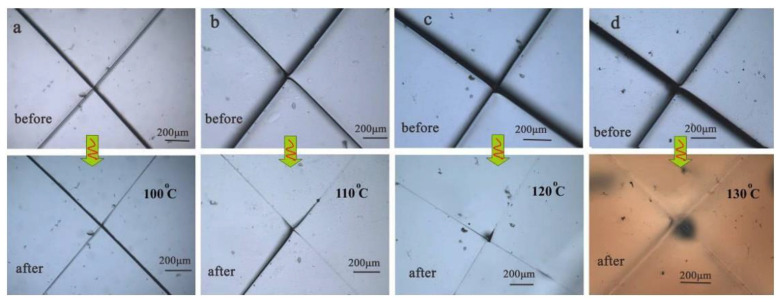
Presents POM images of DAPU films before and after annealing for 5 min at various temperatures: (**a**) 100 °C, (**b**) 110 °C, (**c**) 120 °C, and (**d**) 130 °C [96] (Permission to use was granted by Elsevier).

The paramount property of self-healing polymers is their healing efficiency. The self-healing behavior of Diels–Alder polyurethane (DAPU) samples was evaluated using a POM test in [96]. In this study, self-healing tests were conducted at various temperatures (100, 110, 120, and 130 °C) for 5 min, and POM images corresponding to each condition are depicted in Figure 6. To simulate intentional damage, samples were deliberately incised using a blade. As illustrated in Figure 6a, DAPU exhibited minimal self-healing capability after 5 min at 100 °C, while a slight self-healing effect was observed after the same duration at 110 °C (Figure 6b). Upon increasing the heating temperature to 120 °C, the extensive cracks in DAPU following damage were notably repaired, with only a slim line remaining visible after 5 min (Figure 6c), indicating a high self-healing efficiency. Furthermore, the pre-existing damage in DAPU was almost entirely restored at 130 °C after 5 min (Figure 6d). However, it is noteworthy that the sample exhibited darkening at this elevated temperature. These findings suggest that this furan-maleimide-based polymer necessitates a critical temperature (e.g., 120 °C) to demonstrate its self-healing capability effectively.

### 4.2. Self-Healing in Composites

The development of layered composite materials (sandwich panels) is promising. In such a scheme, each layer performs its specific function and also contains at least one layer with self-healing properties. When assembled, such material is able to minimize damage and quickly restore its original macro-characteristics. Sandwich panels can include various reinforcing components that impart rigidity and stability to the structure, solid, viscous, and liquid fillers, which, when a material defect occurs, react with each other, forming a viscous or solid phase [31,97].

To effectively harness the self-healing effect in layered composite materials, it is essential to incorporate at least one layer capable of fluidity for facilitating mass transfer. One promising candidate meeting this criterion is a borosiloxane (BS)-based material [98,99,100].

**Figure 7:**
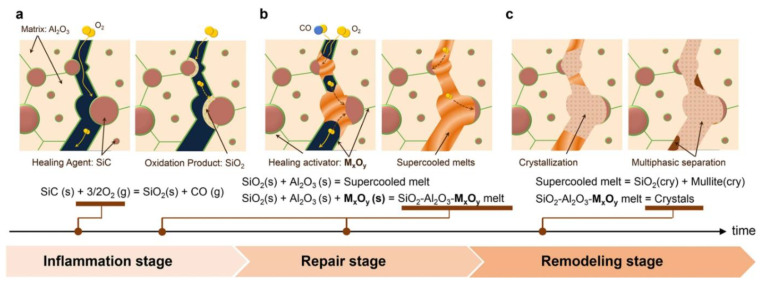
Self-repair mechanisms in Al_2_O_3_/SiC composites and the influence of healing activator networks. (**a**) Oxygen infiltration occurs on cracked surfaces, initiating the oxidation of SiC to SiO_2_, termed the inflammation stage. (**b**) Al_2_O_3_ and M_x_O_y_ dissolve into SiO_2_, creating a mechanically frail, low-viscosity supercooled melt that effectively occupies uneven gaps, referred to as the repair stage. (**c**) Robust crystals begin to nucleate and expand within the supercooled melt, marking the remodeling stage [101] (Figure is available in Open Access).

In [101], the self-repair process underscores the significance of Al_2_O_3_ dissolution into SiO_2_, pivotal for effectively filling gaps with a low-viscosity supercooled melt and for depositing reinforcing crystals essential for complete strength restoration. Drawing inspiration from bone regeneration, the authors have categorized this mechanism into three primary stages: inflammation, repair, and remodeling (Figure 7) Therefore, through the strategic design and integration of a healing activator that fosters these processes, there exists the potential to augment self-healing capabilities further.

**Figure 8:**
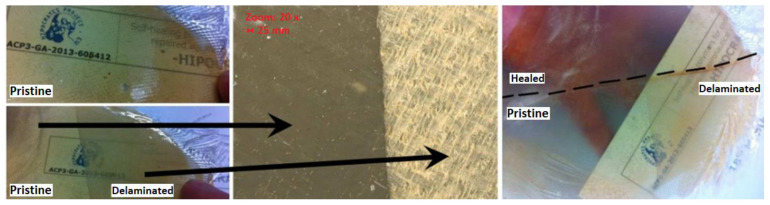
The glass fiber-reinforced epoxy composite functionalized with Diels–Alder undergoes fabrication (**top left**). The woven glass fiber fabrics are separated until halfway into the specimen ((**bottom left**) and microscopic image). Subsequently, half of the damaged area is healed using a hot press (**right**) [102] (The figure is available in Open Access).

Authors of [102] demonstrate the self-healing functionality of a composite film. Half of the sample undergoes artificial damage via manual cleaving of the material stack between the layers of glass fiber fabric. This results in opacity in the damaged area due to reflections from air-fiber and air-resin interfaces occurring at resin cracks and fiber-resin delamination sites (Figure 8). Following this, the damaged substrate is rotated 90 degrees and inserted halfway into the hot press (with the other half sticking out). Pressing at elevated temperatures restores fiber-resin delamination and heals cracks, thereby fully restoring transparency to the sample. Three-quarters of the sample (pristine, pristine-pressed, and pristine-cleaved-pressed) exhibit the same optical appearance, distinctly contrasting with the pristine-cleaved quarter. From this, it can be inferred that self-healing can be successfully achieved without major side effects.

### 4.3. Self-Healing in Ceramics

In addition to polymers, ceramic SHMs are currently being developed. Self-healing ceramic materials often use oxidation reactions, with the volume of oxide exceeding the volume of the starting material. As a result, the products of these reactions, due to the increase in volume, can be used to fill small cracks [103].

The manifestations of self-healing in ceramic materials are not as pronounced or widespread as those observed in polymers. Typically, self-healing in ceramics is limited to addressing small defects, usually on the scale of hundreds of micrometers. However, the phenomenon of “self-ensuring” microcracks, induced by mechanical wear or thermal stress in ceramic materials, offers significant improvements in their operational characteristics [104,105].

**Figure 9:**
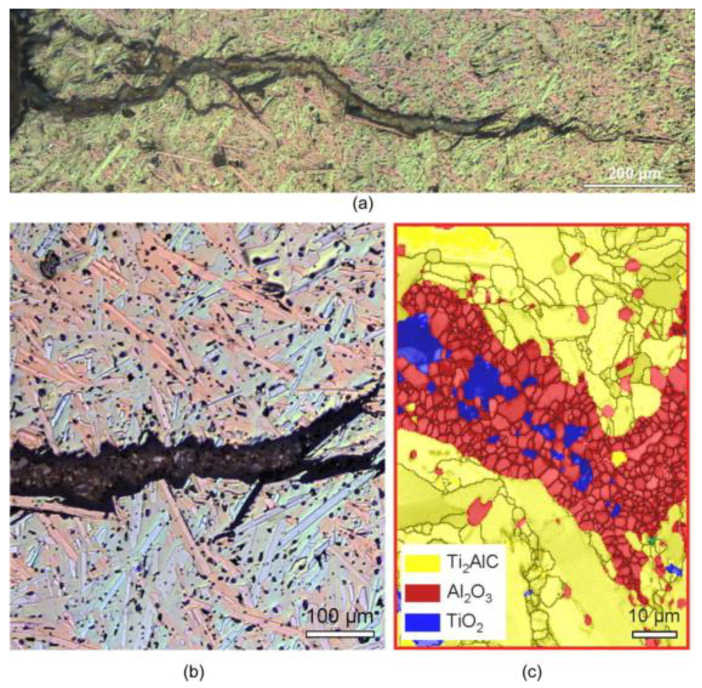
Low- and high-magnification images depict a crack that has been completely filled in the sample following the 8th fracture and subsequent annealing at 1200 °C for 100 h. (**a**) An optical overview image provides a glimpse of the healed crack; (**b**) an enlarged optical image, derived from (**a**), reveals that two opposing fracture surfaces are coated with the same Al_2_O_3_ layer (depicted in black), while the gap between these surfaces is entirely filled with a mixture of Al_2_O_3_ (black) and TiO_2_ (large white particles); (**c**) a detailed micrograph of the healed-damage zone, obtained using scanning electron microscopy with electron backscatter diffraction, offers further insight into the healing process [106] (Permission to use was granted by Elsevier).

For instance, in self-healing Ti_2_AlC ceramics, the mechanism involves the filling of cracks with α-Al_2_O_3_ and TiO_2_, which form at high temperatures in air (see Figure 9) [106].

An additional instance of ceramic “self-healing” is exemplified by the self-healing oxidation observed in SiC ceramics. In this process, active SiC filler particles within the matrix undergo oxidation upon exposure to penetrating oxygen. Consequently, SiO_2_ is formed, effectively filling the crack [107,108].

In contrast, achieving the self-healing effect in metallic materials poses greater challenges due to their unique properties. One such obstacle stems from the nature of the atomic bonds and their limited mobility at operational temperatures. Essentially, defects in metals are rectified through the introduction of more fusible and ductile phases into the primary matrix or via the accelerated formation of agglomerates from phases that precipitate under specific conditions within the base material at defect sites. These melted or precipitated phases have the capacity to fill defects and halt the further propagation of damage. 

### 4.4. Self-Healing in Concrete and Cement

The primary mechanisms underlying the self-healing of cementitious materials can be categorized into three main types: natural or autogenous healing, involving hydration and carbonization reactions; bio-based healing; and activation, achieved through methods such as the application of chemical additives, reactions utilizing fly ash, special expanding agents, and incorporation of geopolymer materials [109,110,111]. 

Collectively referred to as self-healing concrete, these approaches encompass a range of modern developments and innovative solutions aimed at altering material structures to enhance recovery and resistance to various stresses. Given that concrete stands as one of the most ubiquitous materials in the construction and repair industries today, the demand for novel production methods is more pronounced than ever [112]. Notably, researchers at Delft University of Technology, under the leadership of Hank Jonkerson, have conducted experiments on bioconcrete, a material capable of autonomously repairing cracks and damage through the action of embedded bacteria. These experimental studies were conducted to validate theoretical findings and further advance the understanding of self-healing mechanisms in concrete [113,114].

**Figure 10:**
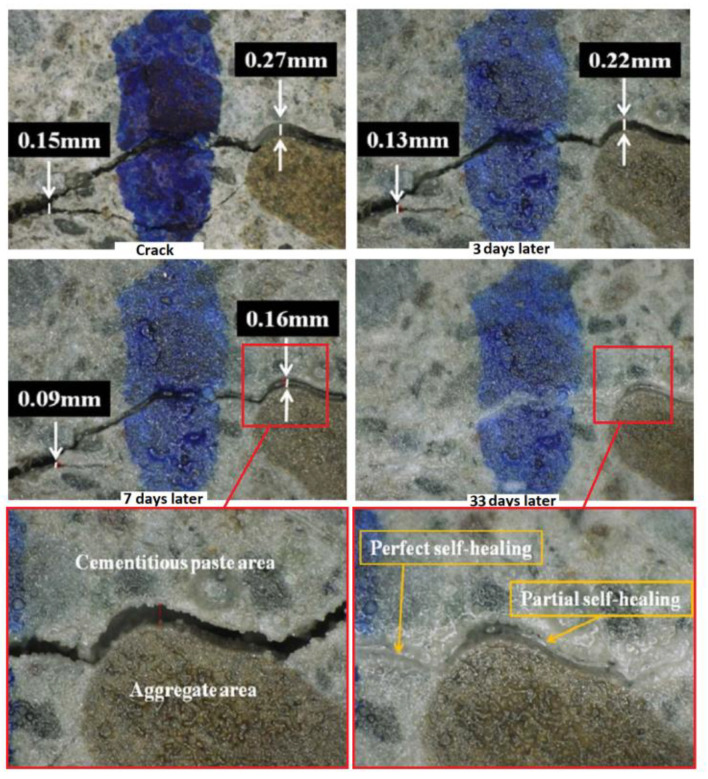
Self-healing of cracks in concrete with mineral fillers [115] (The figure is available in Open Access).

Additionally, recent research [115] demonstrates the self-healing capabilities of cementitious composite materials incorporating geopolymer materials. Figure 10 shows the healing process of cracked self-healing concretes followed similar patterns to conventional concrete when subjected to the same mixing conditions. A crack measuring 0.15 mm in width exhibited self-healing after 3 days of re-curing, as depicted. Subsequently, after 7 days of re-curing, the crack width decreased from 0.22 mm to 0.16 mm. Furthermore, by the 33rd day, it had almost completely self-healed. This recovery process appeared to involve various self-healing phenomena, including swelling, expansion, and re-crystallization. However, it is important to note that cracked aggregates did not self-heal autonomously. Instead, self-healing initially occurred in the cementitious paste area between cracks, facilitating healing between the cementitious paste and the aggregate surface.

A technique has been devised [116] to computationally and experimentally validate the parameters of transition zones surrounding the anti-filtration element (PFE) to facilitate crack self-healing in clay-cement concrete diaphragms (GCBD). By adhering to the prescribed methodology, which includes considerations such as soil granulometry, reverse filter thickness, and layer count, the filtration strength of the system comprising the upper prism, upper transition layer, healing layer, GCBD, lower transition layer, and lower prism can be effectively ensured. The authors propose, in relation to the healing layer, using sandy soil with a particle size of less than 5 mm as a material.

The authors of the article [117] introduced novel techniques aimed at enhancing the strength and self-healing properties of porous composites within concrete structures through both internal and external factors. The method involves two key steps: firstly, establishing a dense and robust material structure, and secondly, incorporating a chemically active mineral, specifically iron sulfide, into the fine filler. This approach facilitates the formation of ettringite-like iron-containing calcium hydrates within the concrete matrix upon cracking, resulting in an expansion of the solid and condensed phases. 

World experience in the development of biomodifying additives includes a fairly wide list of used gram-positive and gram-negative cells (*Sporosarcina pasteurii*, *Bacillus pasteurii*, *B. cohnii*, *B. sphaericus*, *B. pseudofirmus*, *B. cohnii*, *B. halodurans*, *B. subtilis*, *B. megaterium*, *B. alkalinitrilicus*, *Pseudomonas putida*, *Escherichia coli*) [118].

**Figure 11:**
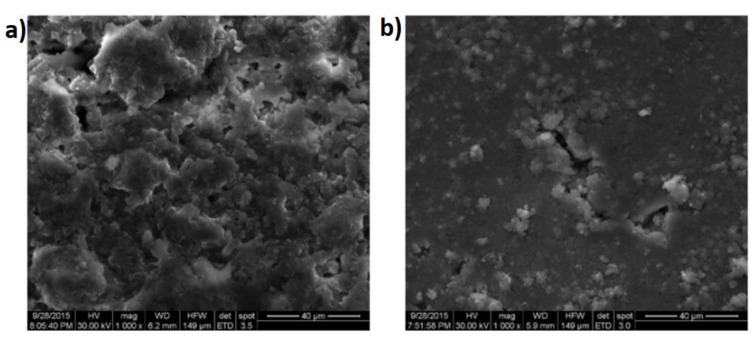
(**a**) The surface of the control sand-cement specimen without the bioadditive at a hardening age of 28 days; (**b**) the surface of the sand-cement specimen with a cell concentration of 0.05% by weight of the cement binder at the same hardening age of 28 days [121]. (The figure is available in Open Access).

The authors in [119] suggest employing ion-plasma treatment to regulate the sorption capacity of natural zeolites intended for use as carriers of active biomodifiers, such as Bacillus pasteurii bacteria, which facilitate self-healing in concrete. The research demonstrates the superior effectiveness of the proposed sorbent modification method over the conventional heat treatment approach. In subsequent studies, findings regarding the capacity of building materials containing Portland cement and gypsum binder to self-repair using capsules containing aerobic bacteria are presented [120]. It was subsequently demonstrated [121] that employing highly porous zeolite as a carrier for an active biomodifier enables the integration of self-healing technology into cement composites. This enables the sealing of defects through the utilization of bacterial byproducts. The enhancement in sample strength is directly linked to the saturation of the microdefect structure within the cement–sand matrix with reinforcing calcium carbonate, as shown in Figure 11.

Employing the developed method of biomodification involving bacteria with urease activity enables the preservation of structural integrity in building constructions, which autonomously repair microstructural defects by sealing them with calcium carbonate at their nascent stages. This biomodification method, aimed at instilling self-healing capabilities, offers numerous advantages, including environmental friendliness, cost-effectiveness, reduced labor requirements for repair and restoration tasks, applicability to structures of varying complexity, and suitability for the restoration of valuable architectural landmarks.

## 5. Key Findings and Future Implications

### 5.1. Key Findings

- Mechanisms of Self-Healing: This review elucidates various mechanisms underlying self-healing processes, spanning from microcapsules and intrinsic chemical reactions to external stimuli-triggered responses. Understanding these mechanisms is fundamental for the development of advanced SHMs.
- Applications Across Industries: SHMs hold immense potential for diverse applications across industries such as implants, prostheses, biomedicine, aerospace, and construction. These materials offer innovative solutions for enhancing durability, safety, and performance in various contexts.
- Material Diversity: This review explores the characteristics and applications of different types of SHMs, including hydrogels, self-healing gels, polymers, ceramics, concretes, and cements. Each material type presents unique advantages and opportunities for advancement.
- Importance of Crack Healing: Crack healing mechanisms play a crucial role in the performance of SHMs, influencing factors such as efficiency and reliability. Understanding and optimizing crack healing processes are essential for maximizing the effectiveness of these materials.

### 5.2. Future Implications

- Tailored Material Design: Future research should focus on developing SHMs tailored to specific applications, considering factors such as environmental conditions, mechanical properties, and biocompatibility requirements.
- Multifunctionality and Integration: There is potential for the integration of self-healing capabilities with other functionalities, such as sensing or antimicrobial properties, to create multifunctional materials with enhanced performance and versatility.
- Sustainable Solutions: Further efforts are needed to explore sustainable sources and manufacturing processes for SHMs, with an emphasis on reducing environmental impact and promoting circular economy principles.
- Translational Research: Continued collaboration between researchers, industry partners, and healthcare professionals is essential for translating advances in SHMs into practical applications, ultimately benefiting society and improving quality of life.

## 6. Conclusions

In conclusion, this review has provided a comprehensive overview of SHMs, covering various aspects including mechanisms, applications, and material types. Throughout this review, we explored the diverse mechanisms of self-healing, ranging from intrinsic chemical reactions to stimuli-triggered processes. Understanding these mechanisms is crucial for the design and optimization of SHMs tailored to specific applications.

We also discussed the wide-ranging applications of SHMs across numerous industries, including biomedical, aerospace, construction, and beyond. From self-healing implants and prostheses in the medical field to self-healing coatings and structures in aerospace and construction, these materials offer innovative solutions for enhancing durability, safety, and performance. Furthermore, we examined the characteristics and applications of various self-SHM types, including hydrogels, self-healing gels, polymers, ceramics, concretes, and cements. Each material type presents unique advantages and challenges, highlighting the importance of tailored approaches for fabrication and optimization. Lastly, we delved into the significance of crack healing mechanisms in SHMs, emphasizing the need to understand factors influencing crack propagation and healing to enhance efficiency and reliability.

Overall, this review underscores the transformative potential of SHMs in addressing a wide range of societal challenges and advancing various industries. By furthering our understanding of self-healing mechanisms and materials, researchers can continue to innovate and develop novel solutions with profound implications for the future.

## Data Availability

Data are contained within the article.
